# Foreign Body Entrapment on the Bearing Surface in Total Hip Arthroplasty: A Report of Three Cases

**DOI:** 10.7759/cureus.76249

**Published:** 2024-12-23

**Authors:** Hisatoshi Ishikura, Kenichi Kato, Atsushi Madachi, Takeyuki Tanaka, Toru Nishiwaki

**Affiliations:** 1 Department of Orthopedic Surgery, Japanese Red Cross Shizuoka Hospital, Shizuoka, JPN; 2 Department of Orthopedics, The University of Tokyo, Tokyo, JPN; 3 Department of Orthopedic Surgery, The University of Tokyo, Tokyo, JPN; 4 Department of Orthopedic Surgery, Tokyo Metropolitan Tama Medical Center, Tokyo, JPN

**Keywords:** bearing surface, entrapment, #foreign body, hips, interposed, total hip arthroplasty: tha

## Abstract

Total hip arthroplasty (THA) is a highly effective surgical intervention for end-stage hip joint disorders. While common complications such as infection, dislocation, and prosthetic loosening are well-documented, rarer complications remain underreported. One such complication is foreign body interposition on the bearing surface, which can compromise joint mechanics and adversely affect outcomes. This study presents three cases of foreign body interposition involving soft tissue, bone fragments, and cement debris, observed postoperatively in patients undergoing THA.

In the first case, a 79-year-old woman presented with soft tissue interposition, which resolved spontaneously through joint motion without surgical intervention. The second case involved a 71-year-old woman with a bone fragment interposed between the femoral head and liner, necessitating reoperation for removal. The third case, a 32-year-old man, required immediate reoperation to remove a 1 cm cement fragment causing a gap on the bearing surface. In all cases, postoperative outcomes were favorable, with patients resuming independent ambulation and reporting no persistent symptoms.

These cases underscore the potential for foreign body interposition to occur during or after THA due to residual debris or displaced tissues. This rare complication has an estimated incidence of 0.2% based on 1,340 procedures at three affiliated hospitals over two years. Key preventive strategies include meticulous removal of debris before reduction, thorough irrigation, and intraoperative imaging to confirm proper alignment. For management, immediate mobilization may resolve soft tissue interpositions, but solid foreign bodies typically require reoperation to prevent long-term damage to the bearing surfaces and subsequent complications, such as osteolysis, implant loosening, or catastrophic ceramic fracture.

This report emphasizes the importance of heightened awareness, careful intraoperative techniques, and prompt postoperative imaging to identify and address this preventable complication. By sharing these insights, we aim to enhance perioperative safety and improve long-term outcomes for patients undergoing THA.

## Introduction

Total hip arthroplasty (THA) is a widely performed and highly effective surgical intervention for patients with end-stage hip joint disorders, offering significant pain relief and improved joint function [1]. Despite its effectiveness, THA can be associated with certain complications. Commonly recognized postoperative complications include bleeding, infection, dislocation, loosening, periprosthetic fractures, deep vein thrombosis/pulmonary embolism (DVT/PE), and nerve palsy [2-4]. These complications are well-documented in the literature and are routinely addressed in surgical planning and perioperative management.

However, there are rare complications that might not be fully understood or commonly reported. One such complication involves the interposition of foreign bodies between the femoral head and acetabular liner. To the best of our knowledge, no reports have described this phenomenon, despite its potential to compromise joint mechanics, cause abnormal wear, and adversely impact patient outcomes. In the context of THA, "foreign bodies" can include not only exogenous materials such as cement debris but also endogenous tissues such as displaced bone fragments or soft tissues.

This study presents three cases of foreign body interposition between the femoral head and liner, with each case involving a distinct type of foreign body: soft tissue, bone fragment, and cement debris. All cases were identified postoperatively and required careful clinical assessment to address the complications. Through detailed descriptions of these cases, we aim to shed light on this overlooked complication, highlight its potential impact on the success of THA, and provide surgical teams with practical insights for prevention and management.

By raising awareness of this rare but significant complication, we hope to enhance the understanding of perioperative risk factors and intraoperative vigilance, ultimately contributing to better patient outcomes in THA.

## Case presentation

Case 1

**Figure 1:**
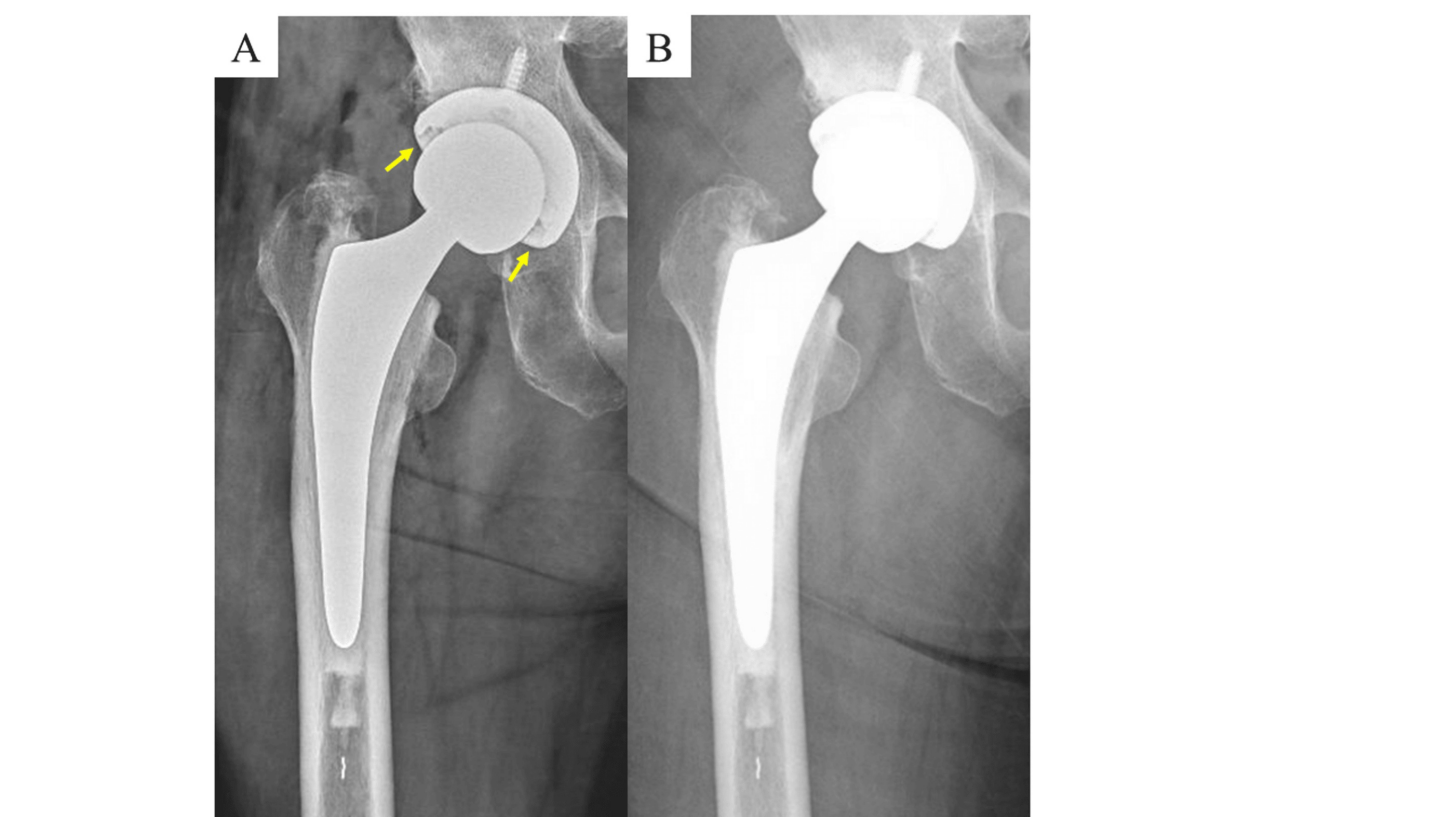
Plain radiographs of the right hip in Case 1. The plain radiograph immediately after total hip arthroplasty (THA) (A) showed a gap in the bearing surface (arrow). The gap had resolved on radiographs taken one week postoperatively (B).

A 79-year-old woman (height: 153 cm, weight: 57 kg) visited a local clinic with a chief complaint of right hip pain persisting for over a year. She was diagnosed with right hip osteoarthritis and underwent conservative treatment, including rehabilitation and analgesics. However, her osteoarthritis gradually progressed. She was diagnosed with end-stage osteoarthritis of the right hip and underwent surgery at our hospital. The surgery was performed using an anterior minimally invasive THA with a leg positioner, and intraoperative fluoroscopy was utilized. The procedure was completed as planned, and there were no abnormalities observed with intraoperative fluoroscopy. However, there was a gap between the femoral head and the liner on the postoperative plain radiograph (Figure 1A).

As no foreign body was observed on the bearing surfaces before reduction, it was suspected that soft tissue might have interposed. A postoperative CT scan revealed no evidence of a solid foreign body on the bearing surfaces, confirming that soft tissue was likely the cause of the interposition. Full weight-bearing walking was initiated the following day, and rehabilitation progressed as planned. There was no worsening of pain after starting rehabilitation. One week later, plain radiographs showed that the femoral head had returned to its original position (Figure 1B). At the one-year follow-up, the patient is walking independently without assistive devices and has shown favorable progress.

Case 2

A 71-year-old woman (height: 151 cm, weight: 46 kg) with dermatomyositis, Sjögren’s syndrome, and rheumatoid arthritis had previously undergone steroid pulse therapy. She developed left hip pain one year earlier, which gradually worsened. She underwent left THA for steroid-induced osteonecrosis of the left femoral head. The surgery was performed in the supine position using the direct anterior approach (DAA) without intraoperative fluoroscopy.

**Figure 2:**
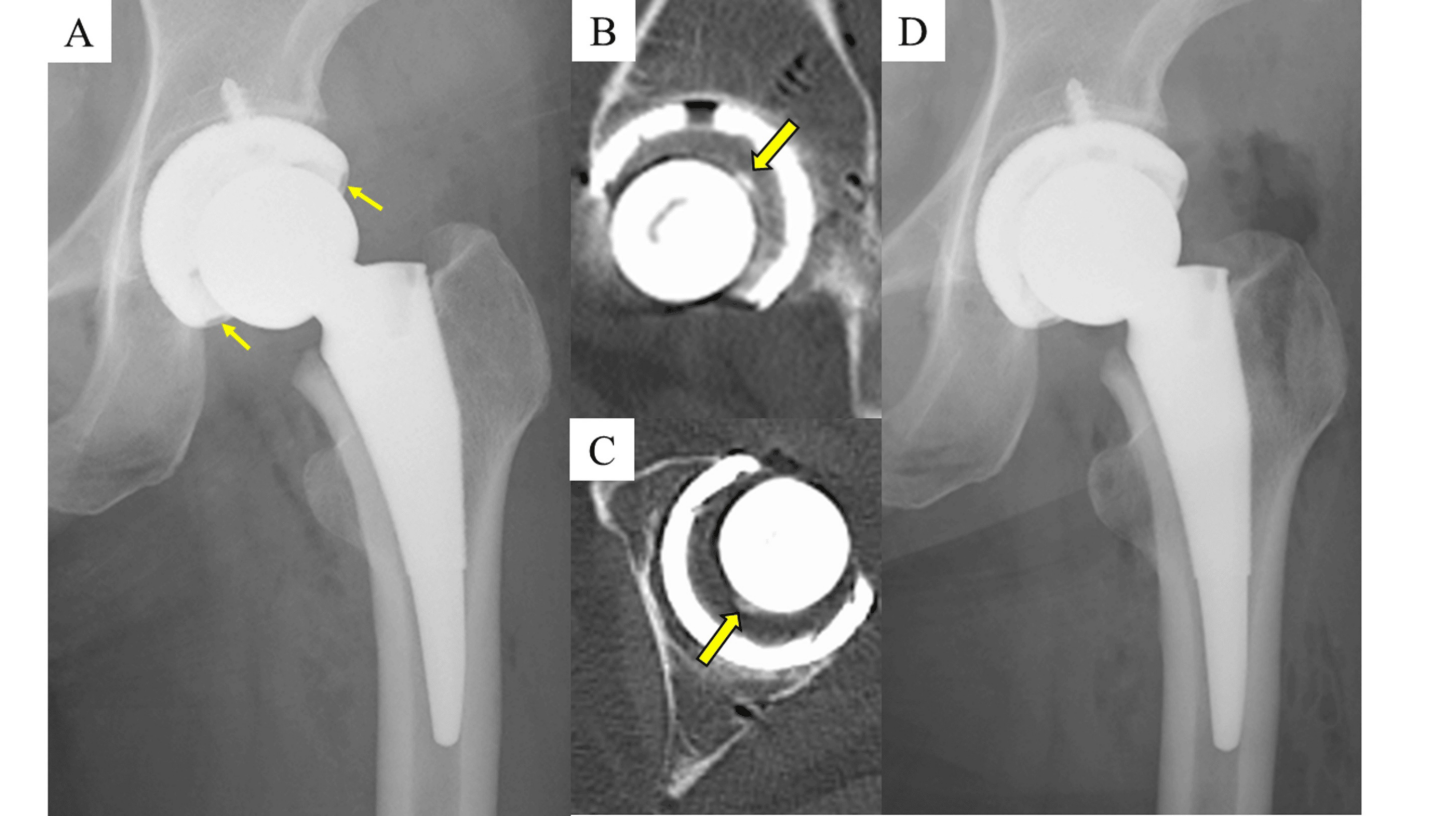
Plain radiographs and CT images of the left hip in Case 2. The plain radiograph immediately after THA (A) showed a gap in the bearing surface (arrow). Sagittal (B) and axial (C) CT images demonstrated a well-defined high-density area (arrow). Postoperative radiograph after removal of the bone fragment (D) showed resolution of the gap in the bearing surface.

Immediately after surgery, plain radiographs revealed that the femoral head was clearly displaced from the liner (Figure 2A). Instead of performing a reoperation immediately, a plain CT scan was obtained later the same day. On sagittal and axial CT images, a well-defined high-density area suggestive of a foreign body was observed between the femoral head and liner (Figure 2B, 2C). A reoperation was performed that evening. During surgery, a small bone fragment was found interposed between the femoral head and liner. After removing the fragment, the femoral head was appropriately seated (Figure 2D). Full weight-bearing walking was initiated the next day, and at the one-year follow-up, the patient is walking stably with a T-cane.

Case 3

A 32-year-old man (height: 178 cm, weight: 82 kg) with a history of thymectomy for myasthenia gravis was diagnosed with steroid-induced bilateral osteonecrosis of the femoral heads. He was initially treated with 60 mg/day of prednisolone, which was tapered to 5 mg/day at the time of surgery. The right femoral head had collapsed with associated osteoarthritic changes, resulting in severe pain and difficulty walking. Therefore, a right THA was performed. The surgery was conducted in the left lateral decubitus position using a posterior approach while preserving the piriformis muscle. A cemented femoral stem was used due to the patient’s near-neutral femoral anteversion.

**Figure 3:**
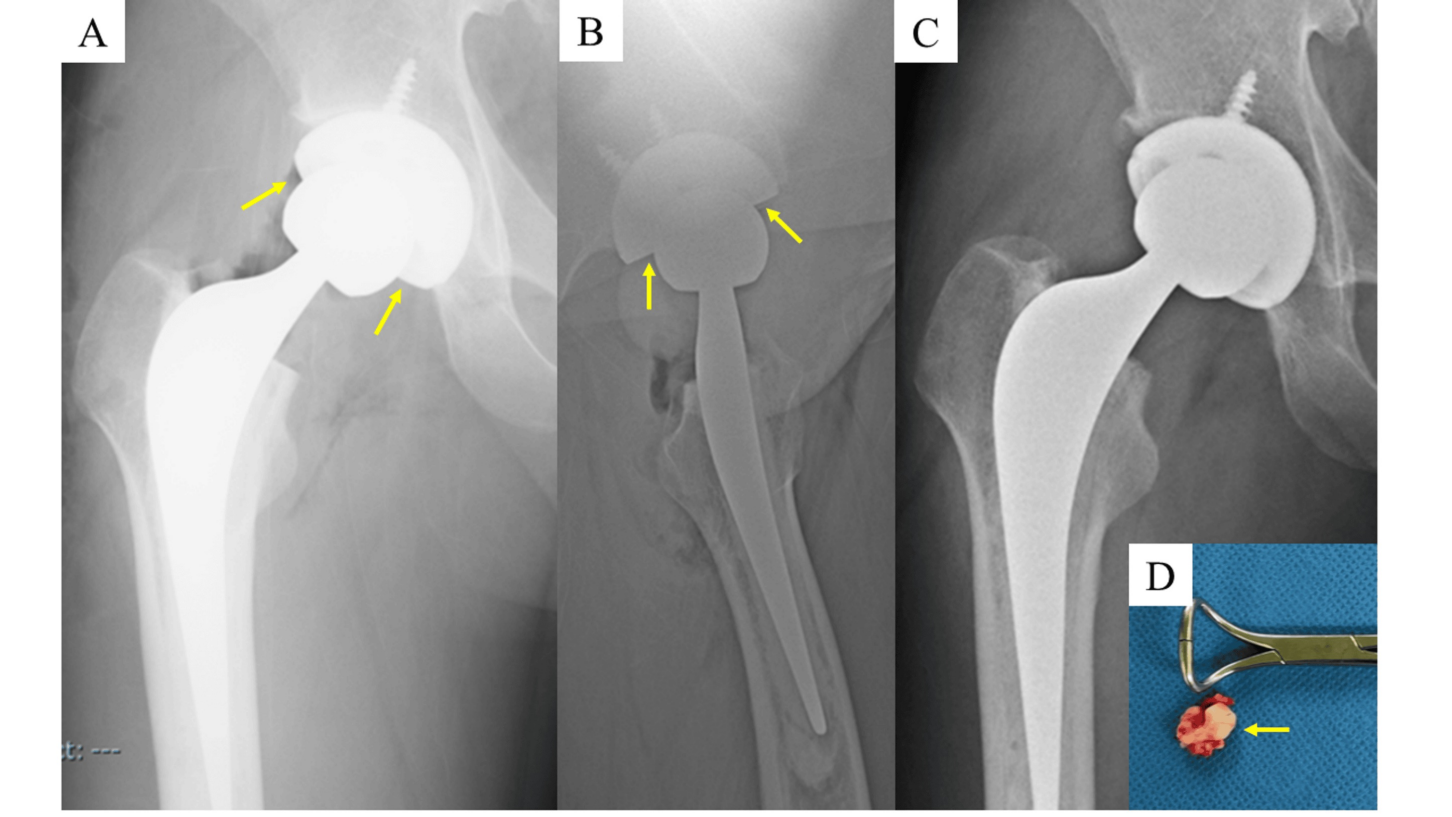
Plain radiographs of the right hip and extracted cement fragment in Case 3. Anteroposterior (A) and axial (B) plain radiographs of the right hip immediately after surgery showed a clear gap in the bearing surface (arrow). The plain radiograph after the removal of the cement fragment (C) showed the resolution of the gap. A cement fragment approximately 1 cm in size was removed from the bearing surface (D, arrow).

Postoperative plain radiographs taken before extubation revealed that the femoral head was clearly displaced from the liner (Figure 3A, 3B). An immediate reoperation was performed, and a cement fragment approximately 1 cm in size was found interposed between the femoral head and liner. After removing the fragment, radiographs confirmed that the femoral head was properly seated (Figure 3C, 3D). Full weight-bearing walking was initiated the following day. At the one-year follow-up, the patient is walking stably without assistive devices and has returned to work.

## Discussion

We report three cases of foreign body interposition in THA. During THA, soft tissues, bone fragments, or cement debris remaining in the surgical field may migrate onto the bearing surface during reduction after implant placement, leading to interposition. As seen in Case 1, if the interposed material is soft tissue such as a portion of the joint capsule or adipose tissue, it may naturally be removed through joint motion. However, in cases where bone fragments or cement debris are involved, waiting for spontaneous removal poses a risk of damage to the bearing surface [5]. If polyethylene is used as the bearing surface, wear debris generated from foreign bodies may cause osteolysis and implant loosening, negatively impacting long-term outcomes [6,7]. If the bearing surface is ceramic, there is a potential risk of catastrophic ceramic fracture [8,9]. For metal-on-metal articulations, wear particles may induce pseudotumor formation and osteolysis, leading to implant loosening [10,11].

To prevent such interposition, the following measures are recommended: (1) removal of any obvious loose debris around the joint before reduction, (2) thorough irrigation of the surgical field before reduction, (3) careful handling of cement to avoid the creation of loose cement fragments, (4) visual inspection of the joint post-reduction to ensure no debris is interposed, (5) intraoperative imaging, such as fluoroscopy, to confirm proper reduction.

Despite these precautions, if foreign body interposition is identified on postoperative plain radiographs, the following steps are recommended. If soft tissue interposition is suspected, joint motion may help resolve the issue. In such cases, attempt mobilization and obtain repeat radiographs to confirm resolution. If the issue persists, or if the interposed material is suspected to be bone or cement fragments, reoperation to remove the foreign body should ideally be performed before extubation. During reoperation, it is crucial to assess the bearing surface for any damage caused by the interposition.

During closed reduction of dislocation after THA, there have been reports of drain tubes or the sciatic nerve becoming interposed [12,13]. Meanwhile, foreign body interposition during THA is not commonly recognized as a typical complication. However, as demonstrated in this report, it often necessitates reoperation, resulting in significant disadvantages for the patient. Considering the preventable nature of this complication with appropriate surgical precautions, it should be acknowledged as a potential risk in THA.

A retrospective investigation of 1,340 THA cases performed at three institutions - Japanese Red Cross Shizuoka Hospital, The University of Tokyo Hospital, and Tokyo Metropolitan Tama Medical Center - between October 2022 and October 2024 identified three cases of foreign body entrapment, as reported in this study. Based on these findings, the estimated incidence of this complication is approximately 0.2%.

This report has certain limitations. Cases involving minor soft tissue interposition that did not result in significant clinical problems may have been overlooked. Additionally, the long-term outcomes of these cases remain unexamined.

Nonetheless, the strength of this report lies in its identification of a rare and previously underreported complication of THA. It serves as a valuable alert for orthopedic surgeons performing hip arthroplasty, highlighting the importance of vigilance to prevent and manage this complication effectively.

## Conclusions

We report three cases of foreign body interposition in THA. Although this complication is rare, it is preventable with appropriate surgical attention. Therefore, it is essential for surgeons performing THA to be aware of this complication and remain vigilant to prevent its occurrence.
